# Carbon stock increases up to old growth forest along a secondary succession in Mediterranean island ecosystems

**DOI:** 10.1371/journal.pone.0220194

**Published:** 2019-07-24

**Authors:** Emilio Badalamenti, Giovanna Battipaglia, Luciano Gristina, Agata Novara, Juliane Rühl, Giovanna Sala, Luca Sapienza, Riccardo Valentini, Tommaso La Mantia

**Affiliations:** 1 Department of Agricultural, Food and Forest Sciences, University of Palermo, Palermo, Italy; 2 Department of Environmental, Biological and Pharmaceutical Sciences and Technologies, University of Campania “L. Vanvitelli”, Caserta, Italy; 3 Landesamt für Natur, Umwelt und Verbraucherschutz Nordrhein-Westfalen, Recklinghausen, Germany; 4 Agrarian and Technological Institute, Peoples’ Friendship University of Russia, RUDN University, Moscow, Russia; 5 Department for Innovation in Biological, Agro-food and Forest systems, University of Tuscia, Viterbo, Italy; Sichuan Agricultural University, CHINA

## Abstract

The occurrence of old-growth forests is quite limited in Mediterranean islands, which have been subject to particularly pronounced human impacts. Little is known about the carbon stocks of such peculiar ecosystems compared with different stages of secondary succession. We investigated the carbon variation in aboveground woody biomass, in litter and soil, and the nitrogen variation in litter and soil, in a 100 years long secondary succession in Mediterranean ecosystems. A vineyard, three stages of plant succession (high maquis, maquis-forest, and forest-maquis), and an old growth forest were compared. Soil samples at two soil depths (0–15 and 15–30 cm), and two litter types, relatively undecomposed and partly decomposed, were collected. Carbon stock in aboveground woody biomass increased from 6 Mg ha^-1^ in the vineyard to 105 Mg ha^-1^ in old growth forest. Along the secondary succession, soil carbon considerably increased from about 33 Mg ha^-1^ in the vineyard to about 69 Mg ha^-1^ in old growth forest. Soil nitrogen has more than doubled, ranging from 4.1 Mg ha^-1^ in the vineyard to 8.8 Mg ha^-1^ in old growth forest. Both soil parameters were found to be affected by successional stage and soil depth but not by their interaction. While the C/N ratio in the soil remained relatively constant during the succession, the C/N ratio of the litter strongly decreased, probably following the progressive increase in the holm oak contribution. While carbon content in litter decreased along the succession, nitrogen content slightly increased. Overall, carbon stock in aboveground woody biomass, litter and soil increased from about 48 Mg ha^-1^ in the vineyard to about 198 Mg ha^-1^ in old growth forest. The results of this study indicate that, even in Mediterranean environments, considerable amounts of carbon may be stored through secondary succession processes up to old growth forest.

## Introduction

### Carbon storage in old growth forests

Forests in the Mediterranean basin have been subject to millennial human exploitation, which deeply modified and simplified their composition, structure, functioning and the ability to provide ecosystem services [1–2]. However, in the last decades, the reduction in the intensity of forest management, and the increase in the protection regime of European forests have allowed the progressive development of structural and biological traits typical of old growth forests (OGFs) [3]. According to FAO [4]: “*An old-growth forest is a primary or a secondary forest which has achieved an age at which structures and species normally associated with old primary forests of that type have sufficiently accumulated to act as a forest ecosystem distinct from any younger age class”*. The ageing without significant human impact is the necessary condition to allow a forest stand to reach an old-growth status in terms of structure, living biomass, deadwood traits and biological diversity [5]. Although OGFs are increasingly studied, most of the research has been focused on aboveground relationships and processes, while letting the belowground level, including soil nutrient dynamics, largely neglected and unexplored. Indeed, the knowledge of the carbon balance of OGFs is limited [6,7], especially in Mediterranean ecosystems [6,8]. Furthermore, although the classical theory by Odum [9] would provide that OGFs are carbon neutral, recent evidence has shown otherwise that they can continue to accumulate carbon in plant biomass and in soils for many centuries before reaching a dynamic steady state [7,10]. Very few studies have been carried out in potentially old-growth Mediterranean oak forests [11,12], including Italy, where the identified OGFs, mostly beech stands, are estimated to cover about 160,000 ha [13]. Although they are likely to be rare, some oak OGF has been reported and investigated in the last few years [14]. However, very little is known about the carbon stocks and belowground pools of these unique ecosystems.

### Carbon dynamics and secondary succession

Even less attention has been paid to the assessment of the carbon variation along a secondary succession up to the latest stage, i.e. the old-growth forest. Only a few studies, mostly carried out in temperate ecosystems of the United States [15,16], compared different successional stages including the OGF. However, such a comparison could be of crucial importance to understand the long-term evolution without human interference of soil organic matter, nitrogen and plant biomass in Mediterranean environments. Here, the widespread abandonment of cultivated lands, due to rapid changes in socio-economic conditions, is expected to bring about large effects on ecosystem C and N pools, with deep ecological implications. Due to their pronounced marginality, Mediterranean island ecosystems have been particularly affected by similar phenomena. The linked secondary succession processes are generally accompanied by the progressive accumulation of carbon and nitrogen in soils, as well as in plant biomass, with very variable patterns depending on the plant species, climatic conditions, and site characteristics [17–19]. For instance, in Sicily (in the Mediterranean basin), the agricultural abandonment alone has produced the accumulation, on average, of SOC stocks of 9.03 Mg ha^-1^ [20]. In Pantelleria (a circum-Sicilian island), we carried out a field experiment in five study sites: a vineyard, three areas characterized by different times since agricultural abandonment (45, 70 and 100 years), and a *Quercus ilex* L. old-growth forest (>105 years). This long time frame provided us with a unique opportunity to improve the understanding of potential carbon accumulation and the evolution of forest ecosystems without human disturbance in Mediterranean islands, where similarly aged oak stands have been rarely found and studied. We aimed at filling this gap of knowledge by investigating the carbon variation in aboveground woody biomass, litter and soil along a chronosequence from vineyard to old-growth forest stage, also assessing the variation in nitrogen content of soil and litter.

## Materials and methods

### Study area

**Fig 1:**
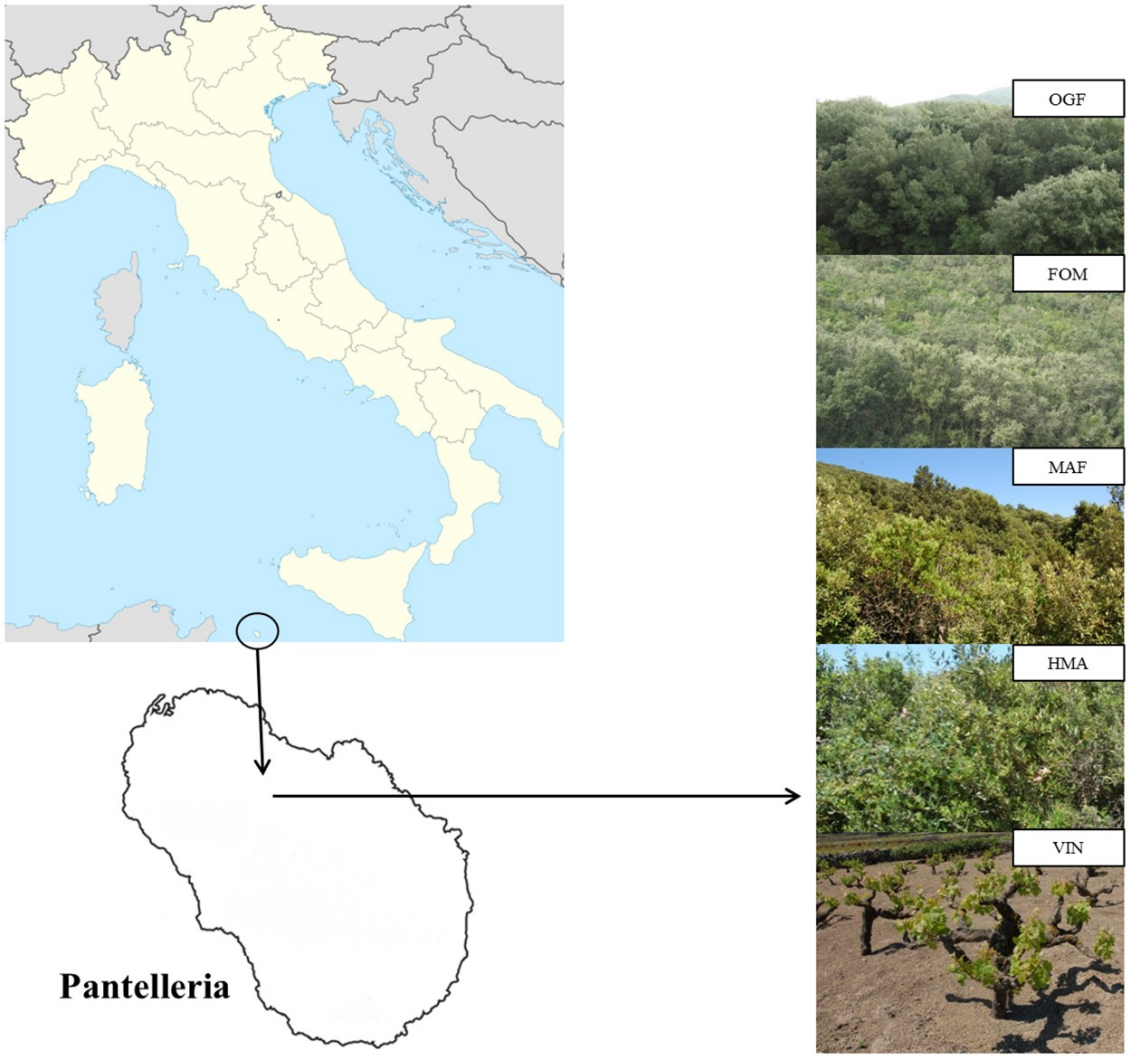
Study areas. Geographical location of Pantelleria island in the Mediterranean, andthe successional stages. OGF: Old-growth forest; FOM: Forest-maquis; MAF: Maquis-forest; HMA: High maquis; VIN: Vineyard.

The study was carried out in Spring 2016 in Pantelleria Island (Italy), situated in the rift of the Sicilian Channel (83 km^2^ area; 36°47′27″N 11°59′38″E) (Fig 1). The island has a typical Mediterranean climate, with most of the precipitation falling between October and February, a mean annual rainfall of 531 mm, and monthly average temperatures ranging from 13.7 °C (January) to 22.1 °C (August) [21]. Until 19th century, most of the reliefs of the island were still covered by high forests, mainly composed by *Quercus ilex*, *Pinus pinaster* Aiton and, more locally, by *Castanea sativa* Mill. [21,22], which were exploited for wood and coal production. After a first period of abandonment, between 1700 and 1800, agriculture, which is carried out on terraces due to the high slope of the territory, had acquired new importance until 1950. The following decades were again characterized by the progressive abandonment of cultivation, and nowadays only about 20% of the original terraces are still cultivated with grapevine, caper, and olive. As a consequence of secondary succession processes, the current landscape of Pantelleria is characterized by patches of old fields differing in the time since abandonment and in the level of woody colonization, determining a high variability in the soil carbon content even at a small scale [23].

### Experimental layout

We carried out a field study following a chronosequence approach where plant successional stages correspond to different time since agriculture abandonment [24]. The study area is localized where still cultivated vineyards and abandoned terraces are present in close proximity to each other. All the environmental factors such as microclimate conditions, geological substrate, soil type, aspect, etc., were homogeneous for all the sampling sites [17,24]. Time since abandonment was identified through the analysis of aerial photos taken by Military Geographical Institute in 1954 and 1968 and by the Regional Land and Environment Department of Sicily in 1987, also using interviews with local farmers. We studied and compared a vineyard (VIN), three successional stages differing in time since abandonment, that is high maquis (HMA: 0–45 years), maquis-forest (MAF: 45–70 years), and forest-maquis (FOM: 70–100 years), and an old-growth forest (OGF: > 105 years) (Fig 1). The HMA is a multi-layered woody formation with a maximum plant height of 2–3 m and a rich understory layer. It is dominated by typical Mediterranean shrub species (*Pistacia lentiscus* L., *Phillyrea latifolia* L., *Calicotome infesta* (C. Presl) Guss. subsp. *infesta*, *Erica arborea* L., *Cistus salvifolius* L.), whereas holm oak is rarely present. The MAF is characterized by dominant holm oak individuals, reaching up to 5 m in height, but it still hosts a considerable presence of shrubs, especially *Pistacia lentiscus* and *Phillyrea latifolia*. The FOM is increasingly dominated by holm oak, with individuals exceeding 5 meters in height, and only some *Pistacia lentiscus* individuals occurring in the understory. The OGF is a pure holm oak stand, about 0.5 hectares extended (stem density: 520 ha^-1^, mean DBH = 41.9 cm, mean height = 12 m), including native lianas typically associated to mature Mediterranean forests such as *Smilax aspera* L. Such forest is one of the oldest of Pantelleria; it includes monumental trees and has probably preserved to obtain wood for building ships. As such forest stand is clearly distinct from younger forest stands, nor it has not been subject to any type of human disturbance for more than one century, it can be considered as an example of Mediterranean OGF. The age of *Quercus ilex* trees was also determined by counting the annual tree rings from eight dominant individuals. Two cores per tree, located at 180° (N-S), were collected with an increment borer (5 mm in diameter) at 1.3 m above the ground. The wood cores were stored and air-dried, for counting tree rings under light, and were sanded to highlight the ring-width of the annual rings. Ring-width was measured to the nearest 0.01 mm, using time series analysis and presentation (TSAP) instrument and software package (Frank Rinn, Heidelberg, Germany).

### Carbon stock in aboveground woody biomass

The aboveground biomass (Mg dry matter ha^-1^) of the vineyard was calculated after oven drying and weighing 10 randomly chosen plants, and considering planting distances of 4 x 4 m (= 0.25 plants m^-2^). For shrub species occurring in HMA, MAF and FOM stages, aboveground living biomass (Mg dry matter ha^-1^) was estimated considering the available literature data about the unitary biomass of each species (see [25] and references therein), and considering their effective cover as derived from local phytosociological relevés [17]. For what concerns the OGF, the volume (m^3^) of the holm oak was obtained using the specific equation fitted in the latest Italian Forest Inventory [26], using the DBH (diameter at breast height, cm) and the height (m) measured in the field. Then, the volume was converted into biomass (Mg dry matter ha^-1^) using the appropriate basic density of holm oak wood (0.579 kg m^-3^) [27]. For all surveyed species, carbon content was derived applying a conversion factor of 0.5 [28]. For the intermediate successional stages (HMA, MAF and FOM), composed by a different relative cover of shrub species and holm oak, the carbon stock was determined through the weighted average of the respective carbon stocks.

### Soil and litter sampling

Soil and litter were sampled according to the protocol of the Italian National Inventory of Forests and Forest Carbon Pools [29], using a chronosequence approach. In each site, after the litter removal, three soil samples were collected at approximately 10-m intervals along a linear transect at two depths (0–15 cm and 15–30 cm), and using the core method [30] for bulk density determination. The litter was collected in a square area of 40 × 40 cm, three times replicate, and distinguished into:

OL horizon, consisting of plant debris, mostly intact, little altered or slightly fragmented leaves, the original form of which is still clearly visible;

OF horizon, consisting of residues of plant tissues finely fragmented and partially decomposed, mixed with fine organic matter.

Overall, 30 soil samples (5 sampling sites x 2 soil depths x 3 replicas), and 12 litter samples (4 sampling sites x 3 replicas) were collected.

### Carbon and nitrogen determination on soil and litter

Carbon and nitrogen content were assessed on 2-mm sieved soil and on both litter types (OL and OF). Organic carbon was determined after the oxidation of OM, under standard conditions, with a solution of potassium dichromate with sulfuric acid [31]. Nitrogen content was determined through the oxidation in sulfuric acid and using hydrogen peroxide to complete mineralization [32].

Soil carbon or nitrogen stock (Mg ha^-1^) was calculated as follows:

C or N stock (Mg ha^-1^) = C or N (g kg^-1^) * soil depth (m) * soil bulk density (g kg^-1^) * 10,000 (m^-2^). In litter, carbon or nitrogen stock (Mg ha^-1^) was calculated starting from the data of litter biomass (kg m^-2^), and considering the effective concentration in litter (in %) of carbon and nitrogen, respectively.

### Statistical analysis

Analysis of variance (ANOVA) was performed to assess the influence of successional stage, soil depth and their interaction on soil organic carbon (SOC), nitrogen and C/N ratio. ANOVA was also performed to assess the influence of successional stage, litter type and their interaction on litter carbon, nitrogen and C/N ratio. Differences between means were tested with the LSD test at P<0.05. SAS statistical program was used [33].

## Results

### Tree age assessment

The number of growth rings is a good estimator of tree age. Trees were sampled at breast height because coring at ground level is more physically difficult and it is not assured to reach the pith in hardwoods like holm oak. Therefore, a regression method was applied to estimate the tree age with height corrections [34]. The oldest holm oak tree was 88 years old (radius of 24 cm), while in the same area a younger tree was 45 years old (radius of 16.1 cm). Adding 15 years to reach the breast height, the oldest tree was estimated to be 103 years old.

### Carbon stock in aboveground woody biomass

The aboveground woody biomass and carbon content considerably increased along the succession (Table 1). Aboveground biomass increased from 30 Mg ha^-1^ (HMA) to more than 200 Mg ha^-1^ (OGF), and carbon stock increased from 15 Mg ha^-1^ (HMA) to more than 100 Mg ha^-1^ (OGF).

**Table 1:** Aboveground biomass and carbon content of woody species in the different successional stages.

Successional Stage	Aboveground woody biomass (Mg ha^-1^)^a^	C stock (Mg ha^-1^)	Holm oak cover (%)
Vineyard	13	6	0
High maquis	30 (20–40)	15	10
Maquis-Forest	70 (60–80)	35	30
Forest-Maquis	110 (100–120)	55	50
Old-growth forest	209	105	100

^a^In brackets the range of estimated values from literature data [25].

### Soil organic carbon and nitrogen

SOC increased along with plant succession from 10.7 g kg^-1^ to 30.2 g kg^-1^ in the more superficial soil layers (0–15 cm), and from 11.2 g kg^-1^ to 15.7 g kg^-1^ in the deeper soil layers (15–30 cm) (Table 2). In the more superficial soil layers, N content increased from 1.2g kg^-1^ (VIN) to more than 3 g kg^-1^ (OGF). The trend of N content in the deeper soil layers was less evident, going from 1.3 g kg^-1^ (VIN) to 1.7 g kg^-1^ (OGF). In the more superficial soil layers, C/N ratio increased from 8.7 to 11.6 going from HMA to FOM, afterwards it decreased. In the deeper soil layers, the ratio remained relatively constant, ranging from 7.6 to 9.0 throughout the succession. In the more superficial soil layers, C stock was more than three times higher in the OGF, going from about 30 Mg ha^-1^ (VIN) to more than 100 Mg ha^-1^ (OGF). Regardless of soil depth, C stock was more than doubled in the OGF. In the more superficial soil layers, N stock was almost three times higher in the OGF, going from about 3.5 Mg ha^-1^ (VIN) to more than 10 Mg ha^-1^ (OGF). Regardless of soil depth, N stock was more than doubled in the OGF.

**Table 2:** Soil carbon and nitrogen content along the secondary succession.

Successional Stage	Soil depth (cm)	C (g kg^-1^)	N (g kg^-1^)	C/N	Soil bulk density (g cm^-3^)	C (Mg ha^-1^)	N (Mg ha^-1^)
Vineyard	0–15	10.7	1.2	8.7	1.00	32.1	3.7
	15–30	11.2	1.3	8.5	1.15	38.7	4.6
High maquis	0–15	11.5	1.3	8.7	1.34	46.4	5.4
	15–30	10.3	1.2	8.8	1.35	41.5	4.7
Maquis-forest	0–15	17.1	1.7	10.1	1.26	64.6	6.4
	15–30	11.5	1.3	8.7	1.34	46.1	5.3
Forest-maquis	0–15	26.5	2.3	11.6	1.16	92.2	8.0
	15–30	11.5	1.5	7.6	1.33	46.1	6.1
Old-growth Forest	0–15	30.2	3.1	9.8	1.14	103.0	10.6
	15–30	15.7	1.7	9.0	1.28	60.3	6.7

Successional stage and soil depth significantly affected soil carbon and nitrogen content and stock but not C/N ratio (Table 3). The interaction successional stage x soil depth was not significant.

**Table 3:** Analysis of variance for soil organic carbon, nitrogen and C/N.

		C_conc_		N_conc_		C/N		C_stock_		N_stock_	
Source of variation	df	F	P	F	P	F	P	F	P	F	P
Successional stage (SS)	4	1.65	0.024	6.34	0.002	0.15	0.963	2.79	0.05	5.00	0.00
Soil depth (Sd)	1	3.83	0.009	8.37	0.009	1.87	0.186	5.46	0.03	4.43	0.05
SS*Sd	4	0.78	0.191	2.08	0.121	0.51	0.727	1.30	0.30	1.49	0.24

### Litter carbon and nitrogen

The biomass of OF litter was considerably higher than OL litter along the succession (Table 4). Both litter types showed an overall increasing trend, but the OF litter reached its maximum value (5.00 kg m^-2^) in the FMA. The relative contribution (in %) of each litter type considerably changed along the succession, with OL litter increasing from 16% to 37%, and OF litter decreasing from more than 83% to less than 63%. Overall, OF litter ranged from 3.8 kg m^-2^ to 4.4 kg m^-2^, whereas OL litter linearly increased from 0.7 kg m^-2^ to 2.6 kg m^-2^. Carbon content decreased in both litter types from about 241 g kg^-1^ to 172 g kg^-1^, and from about 205 g kg^-1^ to 152 g kg^-1^ in OL and OF litter, respectively (Table 4). N content was considerably higher in OF litter from FOM stage onwards, while being similar in VIN and HMA stages. In both litter types, N content showed a generally increasing trend, going from 11.5 g kg^-1^ and 13.7 g kg^-1^ in OL litter, and from 12.3 g kg^-1^ to 16.7 g kg^-1^ in OF litter. The C/N ratio of litter had a strong decreasing trend in both OL and OF. Overall, the ratio was consistently higher in OL litter, where it decreased from almost 21 (HMA) to about 12.5 (OGF), than in OF litter, where it decreased from about 16.5 (HMA) to about 9 (OGF). In the OL litter, C stock was more than two and a half times higher in the OGF, going from 1.7 Mg ha^-1^ (HMA) to 4.4 Mg ha^-1^ (OGF). Conversely, OF litter had an irregular trend, fluctuating from 6.6 Mg ha^-1^ to 7.7 Mg ha^-1^. N stock slightly increased from 0.1 Mg ha^-1^ to 0.4 Mg ha^-1^ in OL litter, and from 0.5 Mg ha^-1^ to 0.7 Mg ha^-1^ in OL litter.

**Table 4:** Litter biomass, carbon and nitrogen content, and C/N ratio in undecomposed (OL) and fragmented litter (OF) along the succession.

Successional Stage	Litter type	Litter biomass (kg m^-2^)	C (g kg^-1^)	N (g kg^-1^)	C/N	C (Mg ha^-1^)	N (Mg ha^-1^)
Vineyard		------	------	------	------	------	------
High maquis	OL	0.73	240.7	11.5	20.9	1.7	0.1
	OF	3.76	205.1	12.3	16.7	7.5	0.5
Maquis-Forest	OL	1.05	213.1	12.8	16.6	2.3	0.1
	OF	4.74	145.6	12.0	12.1	6.8	0.6
Forest-Maquis	OL	1.25	201.9	12.4	16.3	2.5	0.2
	OF	5.01	154.9	15.2	10.2	7.7	0.8
Old-growth forest	OL	2.62	172.3	13.7	12.6	4.4	0.4
	OF	4.36	151.7	16.7	9.1	6.6	0.7

Successional stage significantly affected litter carbon and nitrogen content, as well as C/N ratio, but neither C or N stock (Table 5). Litter type significantly determined litter carbon content, the C/N ratio, C and N stock, but not nitrogen content. The interaction successional stage x litter type was not significant.

**Table 5:** Analysis of variance for litter carbon, nitrogen and C/N.

		C_conc_		N_conc_		C/N		C_stock_		N_stock_	
Source of variation	df	F	P	F	P	F	P	F	P	F	P
Successional stage (SS)	4	15.09	0.000	4.30	0.02	10.46	0.000	0.25	0.86	1.43	0.27
Litter type (L)	1	40.47	0.000	4.09	0.06	20.30	0.000	24.16	0.00	21.23	0.00
SS*L	4	2.18	0.131	1.62	0.22	0.29	0.827	0.79	0.51	0.29	0.83

### Variation of the aboveground carbon balance

**Fig 2:**
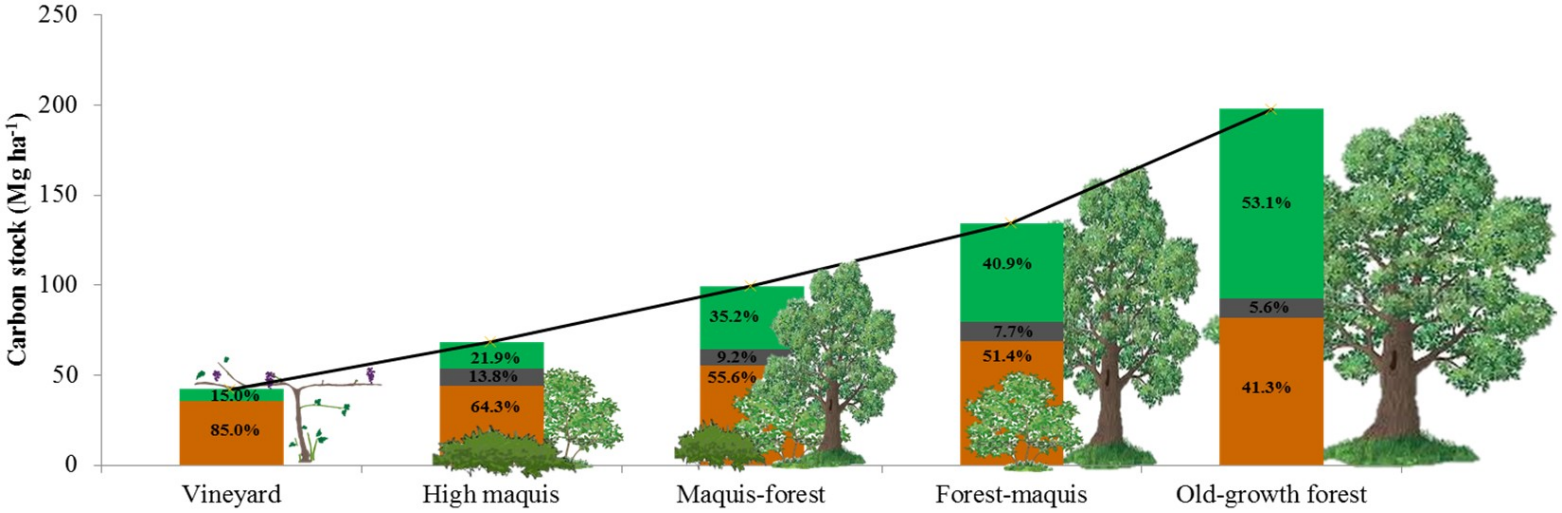
Carbon stock variation. Carbon stock in aboveground woody biomass (in green), litter (in grey), and soil (in orange) in the different successional stages. The relative contribution (in %) of each carbon pool is reported for each successional stage.

Overall, carbon stock in aboveground woody biomass, litter and soil increased from 41.7 Mg ha^-1^ (VIN) to 197.8 Mg ha^-1^ (OGF) (Fig 2). The relative contribution (in %) of each C pool was notably different along the succession, with soil decreasing from 85.0% to 41.3% of the total stock, and aboveground woody biomass, conversely, increasing from 15.0% to 53.1%. The litter contribution was more regular, going from 9.5% to about 11.0% of the total stock. From VIN to OGF stage, carbon stock in woody biomass increased by more than 17 times, whereas in soils it increased by more than 2 times. Nitrogen stock in soil has more than doubled along the succession, increasing from 4.1 Mg ha^-1^ (VIN) to 8.8 Mg ha^-1^ (OGF).

## Discussion

### Carbon stock in aboveground woody biomass

The aboveground C stock (104.8 Mg ha^-1^) of the OGF in Pantelleria island was consistent with what reported in mature, aged or old-growth Mediterranean holm oak forests. This was somewhat unexpected as woodlands in Mediterranean islands have been historically subject to intense and widespread exploitation by man, suffering from presumably higher impacts than continental areas. Such ecosystems store aboveground carbon stocks ranging from 90 to 113 Mg ha^-1^ [35,36], and approach the lower limit of European forests withdrawn from utilization for centuries, showing average values of 130 and 170 Mg ha^-1^ for softwood and hardwood species, respectively [37]. The carbon accumulation of mesophilous oaks such as *Quercus robur* L. and *Quercus petraea* (Mattuschka) Liebl. is intermediate, reaching about 154 Mg ha^-1^ [37]. Therefore, the Mediterranean climatic conditions seem to represent a constraint to the carbon sequestration capacity of forests. Interestingly, the holm oak forest reached an aboveground tree volume (361.8 m^3^ ha^-1^) very close to that found in a Downy oak (*Quercus pubescens* Willd. s.l.) OGF in Sicily (363.9 m^3^ ha^-1^) [12] and in a holm oak OGF in Campania (379.0 m^3^ ha^-1^) [13], under cooler and wetter climatic conditions. Furthermore, as according to ISPRA [38] the holm oak woods have a 1:1 root/shoot biomass ratio, the belowground carbon pool in the OGF in our study may be as large as 100 Mg ha^-1^, determining a carbon stock in total living woody biomass (aboveground plus belowground) of about 200 Mg ha^-1^. The estimates about shrublands are characterized by large uncertainties, with root/shoot ratios ranging from 0.62 [38] to 2.8 [39]. However, even considering the upper limit, the maximum estimated belowground C stocks in the successional stages is about 59 Mg ha^-1^ in FOM.

### Soil organic carbon and nitrogen

Our results showed the progressive increase in SOC and nitrogen content proceeding towards the most mature stages, which is a commonly observed pattern in the ecological successions. This is especially true in OGFs, which host a much higher amount of carbon than younger forests [15], and are characterized by peculiar soil processes, including litter dynamics and nutrient cycling [16,40]. In this study, SOC linearly increased along the succession and has more than doubled from HMA to OGF stage, where it reached 30 g kg^-1^ in the upper soil layers (0–15 cm). Such a value is slightly lower than found in Mediterranean maquis, where SOC ranges from 31 to 36 g kg^-1^ [41], whereas holm oak forests may display a much higher SOC content, approaching 70 g kg^-1^ [42]. Time since agricultural abandonment significantly affected soil N content, which linearly increased along the secondary succession. In the upper soil layers, it almost doubled from HMA to OGF stage, reaching 3 g kg^-1^. Such final value is quite high in the context of Mediterranean holm oak forests, where it ranges from 0.16 g kg^-1^ to 2.9 g kg^-1^ [41,43–45]. In the upper soil layers, the C/N soil ratio slightly increased from 8.7 (HMA) to 9.8 (OGF), thus approximating the lower limit of Mediterranean *Quercus ilex* ecosystems, where this ratio ranges from 11 to about 22 [41,43,44].

### Litter carbon and nitrogen

The main factors affecting the litter traits, such as the plant community, microclimate and decomposer organisms, considerably vary during secondary succession processes. Mayer [15] found more abundant litter and higher decomposition rates in OGFs than in younger forests, with differences attributed to the occurrence of very specialized microbial communities, adapted to deal with a high amount of organic matter (OM) and high soil nutrient fluxes. In our study, the OL litter progressively increased up to the OGF, whereas the OF litter increased from HMA to FOM, but then it decreased in the OGF. The reduction of fragmented litter in the latest stage may be due to two main reasons. Firstly, the litter input in a mature forest may quantitatively exceed the maximum amount of mineralizable OM (quantitative reason). Secondly, plant residues more resistant to mineralization may accumulate (qualitative reason). In Mediterranean thermophilous conditions, the secondary succession is characterized by the progressive establishment of evergreen sclerophyllous species. The sclerophyllous character is expected to reduce litter decomposability, due to the negative relationships between this particular leaf adaptation and leaf N and P content, which, conversely, have a promoting effect on litter decomposition rates [46]. Compared to other Mediterranean woody species, holm oak litter is more stable in time [47] and this could explain the reduction of OF litter in the OGF. For instance, after 3 years of decomposition, cellulose and lignin still represented about 30% of initial OM, compared to just 3–8% in *Myrtus communis* L. [48]. The strong decrease in C/N litter ratio along the succession would suggest the progressive increase in the mineralization rate of OM. A similar decreasing trend has been observed proceeding from old fields to OGFs in the Midwest USA [15]. The C/N litter ratio of holm oak forests, ranging from 46 to 48.9 [44,47], is generally lower than that of the coexisting Mediterranean shrub species, such as *Myrtus communis* and *Phillyrea latifolia*, which have values of 62.0 and 58.6, respectively [44,47]. Hence, the decreasing trend of the C/N litter ratio along the succession could be related to the increasing cover of holm oak.

## Conclusions

In a Mediterranean island, a more than 100 years-long secondary succession significantly increased the carbon stock in aboveground woody biomass, litter and soil. Unlike expectations, the OGF was found to store more than 100 Mg C ha^-1^ in woody biomass, proving a sequestration capacity comparable with aged or mature Mediterranean oak forests of continental areas. Compared to areas subject to ordinary cultivation practices (vineyard), the carbon and nitrogen stock is more than doubled in the latest stage of ecological succession, the old-growth forest. Furthermore, nitrogen soil content reached a quite high level in the context of Mediterranean oak forests. These results do provide novel insights about the maximum potential of soil carbon and nitrogen accumulation following the abandonment of agricultural activities in Mediterranean ecosystems. Indeed, this is one of the very first studies which compared different successional stages up to the final stage. As the abandonment process of cropland and marginal areas is expected to increase in the next future, the carbon stored by woody ecosystems could be greatly enhanced via secondary succession processes. However, further research is needed, also to investigate whether Mediterranean old-growth forests are still actively storing carbon, as suggested by most recent research in other ecosystem types.

## Supporting information

S1 Dataset Soil and litter raw data. (XLSX)
